# Over 1,000 Prescriptions or Favorite Formulæ of Various Authors, Teachers and Practicing Physicians

**Published:** 1899-10

**Authors:** 


Over 1,000 Prescriptions or Favorite Formulæ of Various Authors, Teachers and Practicing Physicians. Second edition, revised and enlarged. Detroit, Mich.: The Illustrated Medical Journal Co. 1899.

This is the second edition of this handy manual, and is just from the press; it has nearly a 100 pages of new matter added. As the practical worth of this kind of a book consists in its having a handy and complete index, this book has it, for some fifteen pages of small type are devoted to this object, and some of the lines have as many as twenty different references to as many different formulæ; this would go to show that there are about 2,000 different prescriptions given in the volume. We notice that many of the newer remedies are among the prescriptions, thus bringing the treatment of many of the diseases down to date. Both old and new writers of both home and foreign countries are represented among its formulæ.

Blank pages are frequently introduced, so that a handy place is furnished for recording any new prescriptions that one might wish to preserve. The printed index will index all such pencilled additions, if care is taken to write them opposite a page with a formula for similar disease; this would then save the bother of indexing the pencilled additions.

